# Primer design and amplification efficiencies are crucial for reliability of quantitative PCR studies of caffeine biosynthetic *N*-methyltransferases in coffee

**DOI:** 10.1007/s13205-018-1487-5

**Published:** 2018-11-01

**Authors:** Simmi P. Sreedharan, Avinash Kumar, Parvatam Giridhar

**Affiliations:** Plant Cell Biotechnology Department, CSIR-Central Food Technological Research Institute, Mysuru, Karnataka India

**Keywords:** Abiotic stress, Caffeine, Internal reference gene, Normalization

## Abstract

Primers having suboptimal amplification efficiencies were shown to falsely represent fold change expression of the *N*-methyltransferases gene family involved in caffeine biosynthesis in *Coffea canephora*. To study this phenomenon, the role of stability of the internal reference gene, as well as the amplification efficiency correction of the primers was investigated. *GAPDH* and *Ubiquitin* exhibited a good stability for studying the ontogeny of endosperm tissue, as well as the leaf transcriptome during stress from salicylic acid, methyl jasmonate, PEG-mediated drought and sudden exposure to light. *Ubiquitin* manifested low variation in Cq under all these stress regimes and in endosperm ontogeny with 30.1–30.9 in the best dataset and 28.8–30.9 in the most deviating dataset. It was observed that problems arising due to improper amplification efficiency of the target or reference genes or both could lead to misinterpretation of gene expression levels. Quantitative RT-PCR performed at a sub-optimal efficiency of GAPDH reference gene at 1.68 led to the faulty interpretation of 2.007 folds upregulation by the 2^−ΔΔCt^ method and 1.705 folds upregulation by Efficiency method for the first *NMT* (Xanthosine methyltransferase), which actually is repressed during dark acclimatization of coffee plants. Efficiency correction improved the reliability of the expression data and also indicated a downregulation of this gene by 0.485 folds and 0.474 folds using 2^−ΔΔCt^ and E method, respectively, in concordance to earlier reports. Hence, efficiency correction of the primers having suboptimal efficiencies is an absolute prerequisite for the accurate calculation of fold change using quantitative RT-PCR.

## Introduction

Caffeine is the most researched molecule from coffee and is designated to a functional role under the ‘Chemical Defense Theory’ (Frischknecht et al. 1985). Nevertheless, there has been much debate about the actual role and the evolution of caffeine in different plant systems. Caffeine accounts for the major purine alkaloid in coffee and is synthesized from the ubiquitous committed precursor, xanthosine, by the **S**alicylic **A**cid **B**enzoic **A**cid **TH**eobromine Synthase, (SABATH) superfamily of methyltransferases (Kato and Mizuno 2004). With the availability of large EST database and complete sequencing of *C. canephora* genome (Denouend et al. 2014) much research needs to now focus on transcriptomics and functional characterization of the coffee genome. Caffeine biosynthetic *N*- methyltransferases are upregulated under influence from light (Kumar et al. 2015a), developmental stage and genotype (Perrios et al. 2015), salicylic acid and methyl jasmonate (Kumar et al. 2015b, 2017). In addition, salinity and drought negatively regulate caffeine content (Kumar et al. 2015c). Future studies on the regulation of caffeine biosynthesis require the assignment of a suitable reference gene for normalization of quantitative PCR.

Quantitative RT-PCR remains a popular tool for transcriptomics due to its ease of application and economic feasibility. Search for appropriate reference gene is indispensable for normalization of qPCR quantification (Kozera and Rapacz 2013). Housekeeping genes like actin, tubulin, GAPDH, ubiquitin, rpl39, and rRNA are routinely used as internal reference genes in different systems (Joseph et al. 2018). However, the reference genes itself vary in their stability and expression level under certain biological conditions. Minimum Information for publication of Quantitative real-time PCR Experiments (MIQE) guidelines (Bustin 2009) recommends the use of most stable reference gene or their combinations for the quantitative analysis of the experimental sets. Technically, it is not feasible to identify a single reference control that can be used for all the experimental sets. Nevertheless, using the statistical programs most suitable combination of reference genes can be selected for each biological experiment sets. The most popular algorithms used for identifying stability of the reference genes include GeNorm (Vandesompele et al. 2002), NormFinder (Andersen et al. 2004), BestKeeper (Pfaffl et al. 2004) and comparative delta-CT (Silver et al. 2006) method, all of which provide a value of stability or normalization factor based on expression of the reference gene in different test and control samples.

Conceptually, relative gene expression by qPCR is calculated by the efficiency method (E method) (Pfaffl 2001), which account for the differences in PCR efficiency of the internal reference gene and target gene and 2^−ΔΔCt^ method that assumes 100% efficiency for both target and reference (Livak 2001). PCR efficiency between samples varies due to dissimilarities in the quantity and quality of cDNA, primer quality, the copy number of transcripts and annealing temperatures. Suboptimal quality of template and primer contribute to errors in calculated fold change due to an appearance of non-stochastic Cq values in the standard curve (Ruijter et al. 2012). PCR efficiency is a critical indicator for the performance of qPCR analysis of multigene families especially involved in secondary metabolism (Arunraj and Samuel 2018). Specificities of primers are not always guaranteed while working with multigene families and the quality of template may vary between samples depending on the impurities in prepared template due to changes in secondary metabolism. Hence, neither 2^−ΔΔCT^ method nor Pfaffl’s E method are necessarily the most accurate description of the actual fold change until efficiencies are optimally corrected. Large-scale qPCR analysis of multigene families requires proper optimization considering all these factors for the generation of reliable data. The present study establishes such errors arising in the quantitation of caffeine biosynthetic NMT gene family in Coffee under light stress and the effect of efficiency correction on the reliability of expression data.

## Materials and methods

### Plant materials and sample preparation

Total RNA was isolated from *Coffea canephora* Pierre ex. Froehner var. robusta cv. S274 leaves of stress treatments (Kumar et al. 2015a, b, 2017) and developing endosperms (Giridhar et al. 2012) and all the primers were adopted from the same studies. Only plant samples from 50 μM salicylic acid, 10 µl methyl jasmonate, 200 mM sodium chloride and 15% (w/v) polyethylene glycol treatments were considered for the present study. Light and endosperm sampling was exactly similar to the method used previously (Kumar et al. 2015a, b). First-strand cDNA synthesis with 1 µg of total RNA was carried out using iScript cDNA synthesis kit (BioRad) with pre-mixed cocktail of Oligo-dT and random hexamer primers. The cDNA preparations from circadian undisturbed and dark acclimatized samples alone were used for the study of fold change.

### Test of stability of internal reference

Stability of the reference genes, *GAPDH* and *Ubiquitin*, were carried out by comparing Cq values in control and treated samples of stressed plants and in the ten different growth phases of endosperms. Twofold dilutions of control leaf and endosperm cDNA (1, 1:2, 1:4, 1:8, 1:16, 1:32 and 1:64 dilutions) was used to generate standard plots of the reference genes and the Cq was corrected using the formula c2 = c1*log(a1)/log(a2) where c2 is the corrected Cq value, if the sample would have amplified with the efficiency equaling a2. For leaf samples, the Cq of *Ubiquitin* was corrected according to the efficiency of *GAPDH* and in ontogeny the Cq of *GAPDH* was corrected according to that of *Ubiquitin*. Amplification curves obtained for the experimental setup using 1:15 diluted cDNA as a template was analyzed using RefFinder program (http://leonxie.esy.es/RefFinder/). qPCR was performed in Applied Biosystems Quantstudio5 instrument using Ssofast Evagreen master mix (BioRad) supplemented with ROXII (Takara biosciences) with following parameters: initial denaturation of 95 °C for 30 s, followed by 40 cycles of denaturation at 95 °C for 5 s and annealing/extension at (58 °C) for 30 s.

### Standard curve for efficiency calculation

Standard plot was made from a dilution series of 1:5, 1:10, 1:20 and 1:40 dilutions of mixed cDNA pooled from all the stress samples. Reactions were repeated in three different annealing temperatures (55 °C, 57 °C and 59 °C). qPCR reactions were carried out in Applied Biosystems Quantstudio 5 instrument with Ssofast Evagreen master mix (BioRad) supplemented with ROXII (Takara biosciences). The cycle parameters included initial denaturation of 95 °C for 30 s, cycle denaturation at 95 °C for 5 s and annealing/extension at (55–59 °C) for 30 s (40 cycle). Efficiency of reaction was calculated from the slope using the formula *E* = 10^−1/slope^. Fold change between cDNA of circadian undisturbed and dark acclimatized plant samples for XMT and MXMT genes was calculated using the method of Pfaffl (2001), ${\text{ratio}}\;{\text{=}}\;\frac{{{{\left( {{E_{{\text{target}}}}} \right)}^{\Delta {\text{C}}{{\text{P}}_{{\text{target}}}}\;({\text{control}} - {\text{target}})}}}}{{{{\left( {{E_{{\text{ref}}}}} \right)}^{\Delta {\text{C}}{{\text{P}}_{{\text{ref}}}}\;({\text{control}} - {\text{target}})}}}}$ and 2^−ΔΔCt^ method (Livak 2001).

## Results and discussion

### Stability of Glyceraldehyde 3-phosphate dehydrogenase (GAPDH) and Ubiquitin internal reference genes

**Fig. 1:**
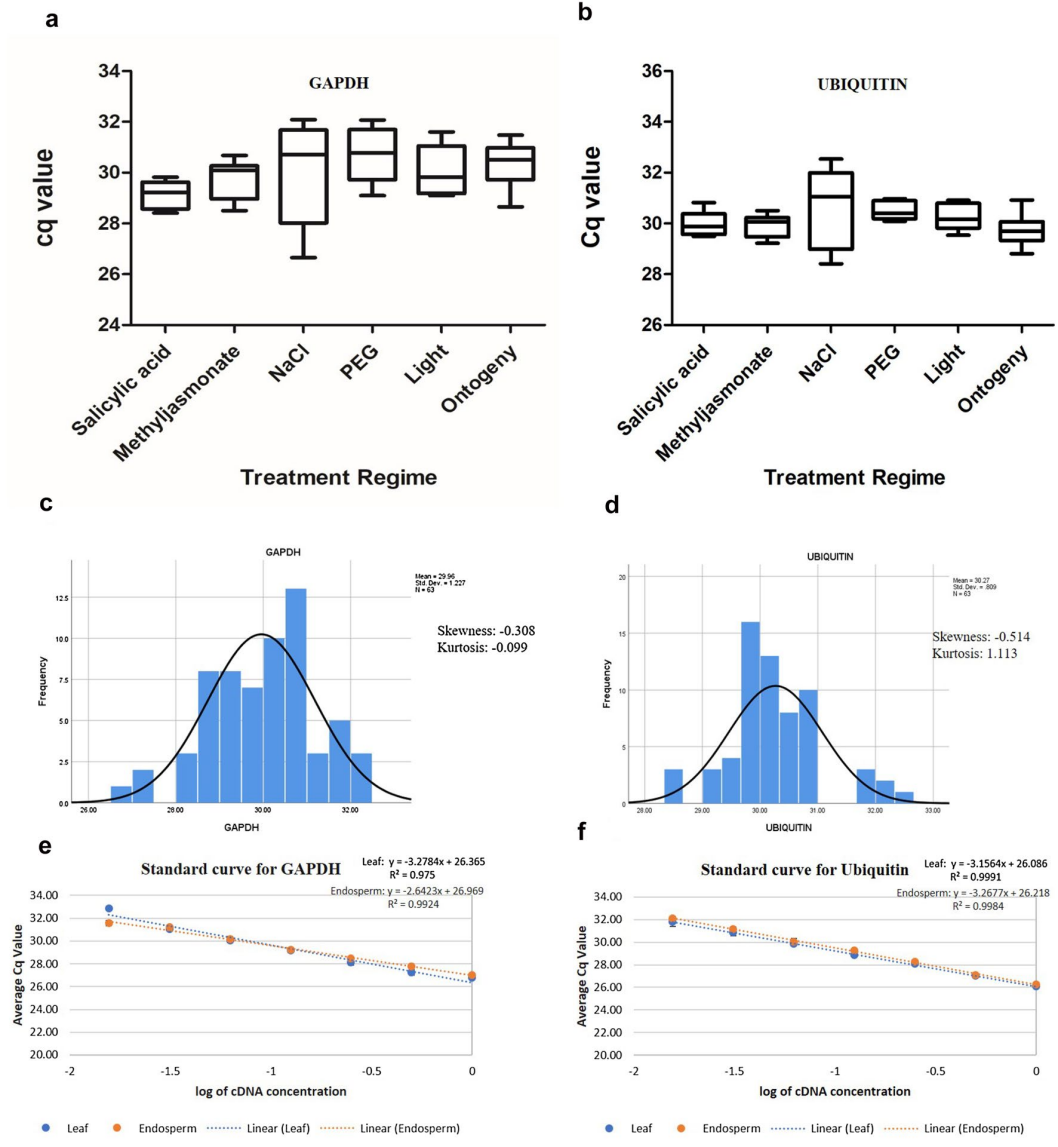
Variation in internal reference genes and their standard curves. Box plot depicting the variation of Cq of (**a**) GAPDH and (**b**) Ubiquitin under different experimental conditions of abiotic stress and ontogeny in *Coffea canephora*; Cq values showing normal distribution for (**c**) GAPDH and (**d**) Ubiquitin; standard curve plotted from different dilutions of leaf and endosperm cDNA for (**e**) GAPDH and (**f**) Ubiquitin

Most suitable internal reference gene for qPCR by SYBR chemistry has been identified for coffee to study rust infection (Vieira et al. 2011), coffee berry disease (Figueiredo et al. 2013), embryogenic tissue (Freitas et al. 2017), water stress (Fernandes-Brum et al. 2017) and chilling stress (Goulao et al. 2011). *GAPDH* and *Ubiquitin* are considered as the most stable internal reference genes combination for qPCR expression studies during water stress and *GAPDH* for studies on fruit ontogeny (Barsalobres-Cavallari et al. 2009; Cruz et al. 2009). In the present study, the stability of both the internal reference gene was tested under conditions of light exposure, salicylic acid, methyl jasmonate, salinity and drought stress on leaf cDNA and during development in endosperm tissues. A box plot graph indicates the higher power of *Ubiquitin* as a reference gene in the analysis between control and stress treated samples from leaves of salicylic acid, methyl jasmonate, PEG-mediated drought and light exposed plants as well as in developing endosperms (Fig. 1a, b). Neither *GAPDH* nor *Ubiquitin* were suitable for studying salinity stress. Strictly essential genes like EF1 and EF1α were predicted to be suitable reference genes for salinity stress in another study on *C. arabica* (Carvalho et al. 2013). The frequencies of Cq values for control and treated samples of leaf and for developing endosperms distributed normally (Fig. 1c, d). Standard curves also indicated that *Ubiquitin* has more power in studying the difference in gene expression between leaf and endosperm tissues (Fig. 1e, f). The amplification curves could be most suitably used at dilutions of 1:4 to 1:32 for both *GAPDH* and *Ubiquitin*.

Stability values were obtained for *GAPDH* and *Ubiquitin* in intra and inter-group comparisons using comparative delta CT, Bestkeeper, NormFinder and GeNorm encompassed in the RefFinder package. The data described in Table 1 indicated that stability values improve by use of efficiency corrected Cq. This difference in efficiency could affect the range of fold change while using these two reference genes or while comparing a reference with the target. M-value of the stress subset was 0.760 in GeNorm analysis of the uncorrected Cq values. However, the M-value improved to 0.039 after efficiency correction of Cq indicating *GAPDH* and *Ubiquitin* to be a stable combination of reference for fold change calculation involving stress experiments. Addition of the endosperm dataset to the consolidated data, disrupted the stability value (*M* = 0.856 for uncorrected and *M* = 3.240 for corrected). Stability values for *GAPDH* and *Ubiquitin* using NormFinder was lowest for salicylic acid treatment (0.009) and highest for endosperm ontogeny (0.053). RefFinder analysis also indicated that the average standard deviation with delta CT method reduced upon Cq correction in individual subsets as well as ‘treatment alone’ consolidated data, whereas, an opposite was observed on the total combined stress and endosperm dataset. Moreover, the uncorrected Cq values for certain subsets varied at > 1.0 values. Hence, 2^−ΔΔCT^ would not be an appropriate method to calculate fold change using these reference genes. Also, correcting Cq for reference genes reduced the standard deviations of ΔCt observed for different datasets.

**Table 1:** Stability of Glyceraldehyde 3-phosphate dehydrogenase and *Ubiquitin* reference genes in different experimental datasets

Group	Gene	RefFinder comprehensive	Delta Ct	BestKeeper		NormFinder	GeNorm
				SD ± Cq	CV ± [%]		
Efficiency corrected Cq							
SA	UBI	1.00	0.02	0.47	1.66	0.009	–
	GAPDH	1.68	0.02	0.48	1.66	0.009	–
	GAPDH/UBI	–	–	–	–	–	0.018
MEJ	UBI	1.00	0.02	0.62	2.19	0.012	–
	GAPDH	1.68	0.02	0.65	2.19	0.012	–
	GAPDH/UBI	–	–	–	–	–	0.025
NaCl	UBI	1.00	0.06	1.27	4.34	0.029	–
	GAPDH	1.68	0.06	1.31	4.34	0.029	–
	GAPDH/UBI	–	–	–	–	–	0.059
PEG	UBI	1.00	0.04	0.84	2.82	0.019	–
	GAPDH	1.68	0.04	0.87	2.82	0.019	–
	GAPDH/UBI	–	–	–	–	–	0.037
Light	UBI	1.00	0.03	0.83	2.90	0.017	–
	GAPDH	1.68	0.03	0.86	2.90	0.017	–
	GAPDH/UBI	–	–	–	–	–	0.034
Ontogeny	UBI	1.19	0.11	0.41	1.38	0.053	–
	GAPDH	1.41	0.11	0.33	1.38	0.053	–
	GAPDH/UBI	–	–	–	–	–	0.106
Consolidated	UBI	1.00	3.24	0.83	2.86	1.620	–
	GAPDH	1.68	3.24	2.69	9.64	1.620	–
	GAPDH/UBI	–	–	–	–	–	3.240
Consolidated minus ontogeny	UBI	1.00	0.04	0.92	3.19	0.019	–
	GAPDH	1.68	0.04	0.95	3.19	0.019	–
	GAPDH/UBI	–	–	–	–	–	0.039
Efficiency Un-corrected Cq							
SA	UBI	1.00	0.40	0.35	1.15	0.201	–
	GAPDH	1.68	0.40	0.48	1.66	0.201	–
	GAPDH/UBI	–	–	–	–	–	0.401
MEJ	UBI	1.00	0.44	0.34	1.13	0.219	–
	GAPDH	1.68	0.44	0.64	2.15	0.219	–
	GAPDH/UBI	–	–	–	–	–	0.437
NaCl	UBI	1.19	0.77	1.35	4.39	0.386	–
	GAPDH	1.41	0.77	1.31	4.34	0.386	–
	GAPDH/UBI	–	–	–	–	–	0.772
PEG	UBI	1.00	0.95	0.33	1.09	0.475	–
	GAPDH	1.68	0.95	0.87	2.82	0.475	–
	GAPDH/UBI	–	–	–	–	–	0.950
Light	UBI	1.00	0.72	0.41	1.35	0.359	–
	GAPDH	1.68	0.72	0.86	2.90	0.359	–
	GAPDH/UBI	–	–	–	–	–	0.719
Ontogeny	UBI	1.00	0.71	0.41	1.38	0.353	–
	GAPDH	1.68	0.71	0.65	2.13	0.353	–
	GAPDH/UBI	–	–	–	–	–	0.706
Consolidated	UBI	1.00	0.86	0.55	1.83	0.428	–
	GAPDH	1.68	0.86	0.88	2.95	0.428	–
	GAPDH/UBI	–	–	–	–	–	0.856
Consolidated minus ontogeny	UBI	1.00	0.74	0.57	1.87	0.368	–
	GAPDH	1.68	0.74	0.95	3.19	0.368	–
	GAPDH/UBI	–	–	–	–	–	0.736

### Effect of efficiency correction on data analysis of fold change

**Fig. 2:**
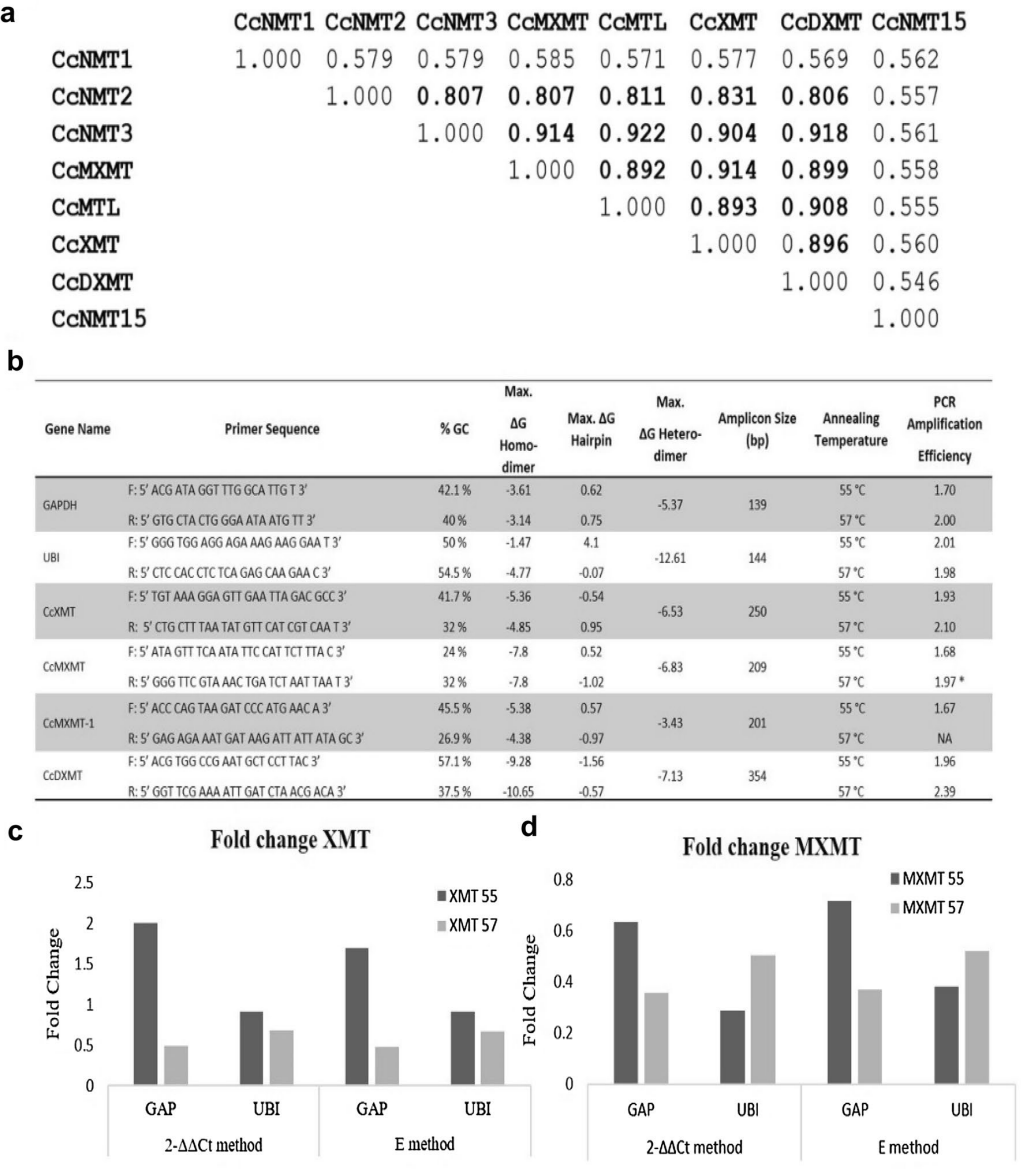
Effects of primers quality and specificity on fold change of the homologous caffeine biosynthetic genes. **a** Sequence similarity in *C. canephora* NMTs; **b** properties of primers; fold change calculated using 2^−ΔΔCT^ method and E method with consistent and inconsistent efficiencies of target and reference gene, **c** fold change for XMT gene, **d** fold change for MXMT gene

Quantitative RT-PCR analysis of transcripts of multigene families, especially genes involved in secondary metabolism, suffer due to issues relating to the specificity of primer and purity of isolated RNA. *C. canephora* NMTs share greater than 80% similarity (Denoued et al. 2014), an example of which is depicted in Fig. 2a. The primers used in this study were designed based on EST sequences prior to the publication of the coffee genome (Fig. 2b). Though the primers amplified specific genes, the *CcMXMT1* forward and *CcDXMT* reverse primer has a base substitution at 10th /24 and 18th /24 nucleotide position from the 5´end of the primer, respectively. Also, the *MXMT* reverse primer shows cross specificity to *CcMTL1* and the *CcDXMT* reverse primer to *CcMTL* and *CcMXMT*—both of which could effectively alter their PCR efficiencies and its dynamics under different experimental datasets. Additionally, the primer binding site on the genome are slightly prone to contain SNPs and hence may have single mismatches depending of genotypes. Also, the primer pairs had varying amplification efficiencies at different temperatures. For *GAPDH*, efficiencies are 1.68 (55 °C), 2.01 (57 °C) and 1.84 (59 °C). *Ubiquitin* amplified with more consistent efficiencies of 2.01, 1.98, 1.98, respectively at these temperatures. XMT primers amplified at efficiencies of 1.93 (55 °C) and 2.10 (57 °C) whereas, MXMT primers at 1.68 and 1.97 and DXMT primers at 1.96 and 2.39, respectively. MXMT1 primers worked only at 55 °C with 1.67 efficiency. It was noted that primers that had optimal design according to Coffee genomic sequence, for example, *XMT* and *Ubiquitin*, did not vary much in efficiency at different annealing temperatures. However, the sub-optimally designed *GAPDH* and *MXMT* exhibited higher difference in efficiency at a slight change of annealing temperature.

A minute repression of caffeine and NMT expression is observed in the leaves of dark acclimatized plants of coffee when compared to circadian undisturbed plants (Kumar et al. 2015a). Hence, these samples were used to study the effect of amplification efficiencies on fold change calculation. The calibrator DNA was prepared from pooled samples of a larger dataset as mentioned in material and methods. The fold change was calculated by the 2^−ΔΔCt^ method (Livak 2001) and the E method (Pfaffl 2001), with consideration of correction under optimal PCR annealing temperatures. *XMT* and *Ubiquitin* genes amplify with good efficiency at both 55 °C and 57 °C. Fold change in *XMT* using *Ubiquitin* reference gave comparable results using 2^−ΔΔCt^ method (0.915 fold) and E method (0.913 fold) at 55 °C and efficiencies of 1.89 and 1.99. Fold change calculation by efficiency correction using standard plot at annealing temperature of 57 °C was also comparable between 2^−ΔΔCt^ method (0.680 fold) and E method (0.668 fold). However, at suboptimal temperature for *GAPDH* at 55 °C, the calculated fold change was different between 2^−ΔΔCt^ method (2.007 fold) and E method (1.705 fold). Furthermore, it is known that the XMTs are downregulated when circadian undisturbed plants are subjected to dark conditions (Kumar et al. 2015a). It is interpreted that at suboptimal efficiencies of reference gene, neither the 2^−ΔΔCt^ method nor *E* method were able to correct an erroneous upregulation plotted for XMT target gene. However, the efficiency of *GAPDH* and *XMT* corrected by standard curve plot at 57 °C, markedly reduced the fold change to 0.485 folds and 0.474 folds by 2^−ΔΔCt^ and *E* method, respectively. Upon correction of efficiency, the fold change calculated for *XMT* with *GAPDH* became comparable to the fold change calculated using *Ubiquitin*.

*MXMT* amplification is prone to relaxed specificity from the reverse primer and also the primer set work at low efficiency at 55 °C as similar to *GAPDH*. Performing efficiency corrections at 57 °C led to more consistency in fold change comparisons using the different methods of 2^−ΔΔCt^ and *E* method as for *XMT*. However, the dynamics of fold change were different when comparing between different reference genes.

## Conclusions

*GAPDH* and *Ubiquitin* genes have low variability between control samples and treatments involving salicylic acid, methyl jasmonate, light exposure, PEG and ontogeny of endosperms with correction of their efficiencies. Primer efficiency is crucial for reduction of errors in fold change calculations of caffeine biosynthetic NMTs. Efficiency correction by qPCR standardization overcomes the errors in the range of fold changes. Hence, we reiterate the importance of efficiency calculations of individual primers as inevitable prior to expression studies. In addition, this could reduce the chances of falsely interpreting overexpression for genes that may actually be repressed or to prevent exaggeration of fold changes.

## References

[CR1] Andersen CL, Jensen JL, Orntoft TF (2004). Normalization of real-time quantitative reverse transcription-PCR data: a model-based variance estimation approach to identify genes suited for normalization, applied to bladder and colon cancer data sets. Cancer Res.

[CR2] Arunraj R, Samuel MA (2018). Integration of amplification efficiency in qPCR analysis allows precise and relative quantification of transcript abundance of genes from large gene families using RNA isolated from difficult tissues. Brief Funct Genomics.

[CR3] Barsalobres-Cavallari CF, Severino FE, Maluf MP, Maia IG (2009). Identification of suitable internal control genes for expression studies in *Coffea arabica* under different experimental conditions. BMC Mol Biol.

[CR4] Bustin SA, Benes V, Garson JA, Hellemans J, Huggett J, Kubista M, Mueller R, Nolan T, Pfaffl MW, Shipley GL, Vandesompele J, Wittwer CT (2009). The MIQE guidelines: minimum information for publication of quantitative real-time PCR experiments. Clin Chem.

[CR5] Cruz F, Kalaoun S, Nobile P, Colombo C, Almeida J, Barros MG, Romano E, Grossi-de-Sa MF (2009). Evaluation of coffee reference genes for relative expression studies by quantitative real-time RT-PCR. Mol Breed.

[CR6] de Carvalho K, Bespalhok Filho JC, dos Santos TB, de Souza SGH, Vieira LGE, Pereira LFP, Domingues DS (2013). Nitrogen starvation, salt and heat stress in coffee (*Coffea arabica* L.): Identification and validation of new genes for qPCR normalization. Mol Biotechnol.

[CR7] Denoeud F, Carretero-Paulet L, Dereeper A, Droc G, Guyot R, Pietrella M, Zheng C, Alberti A, Anthony F, Aprea G (2014). The coffee genome provides insight into the convergent evolution of caffeine biosynthesis. Science.

[CR8] Fernandes-Brum CN, Garcia B, Moreira R, Sagio SA (2017). A panel of the most suitable reference genes for RT-qPCR expression studies of coffee: screening their stability under different conditions. Tree Genet Genomes.

[CR9] Figueiredo A, Loureiro A, Batista D, Monteiro F, Várzea V, Pais MS, Gichuru EK, Silva MC (2013). Validation of reference genes for normalization of qPCR gene expression data from *Coffea spp*. hypocotyls inoculated with *Colletotrichum kahawae*. BMC Res Notes.

[CR10] Freitas NC, Barreto HG, Fernandes-Brum CN, Moreira RO, Chalfun-Junior A, Palva LV (2017). Validation of reference genes for qPCR analysis of *Coffea arabica* L. somatic embryogenesis-related tissues. Plant Cell Tiss Organ Cult.

[CR11] Frischknecht PM, Ulmer-Dufek J, Baumann TW (1985). Purine alkaloids formation in buds and developing leaflets of Coffea arabica: expression of an optimal defence strategy?. Phytochemistry.

[CR12] Giridhar P, Kumar A, Simmi PS (2012) Differential expression of WRKY transcriptional factors in endosperm tissues during stress and ontogeny of fruits of *Coffea canephora* with respect to caffeine biosynthesis. In: Proceedings of 24th International Conference on Coffee Science (ASIC), Sanjose, Costa Rica. pp. 522–526

[CR13] Goulao LF, Fortunato AS, Ramalho JC (2011). Selection of reference genes for normalizing quantitative Real-Time PCR gene expression data with multiple variables in *Coffea* spp. Plant Mol Biol Report.

[CR14] Joseph JT, Poolakkalody NJ, Shah JM (2018). Plant reference genes for development and stress response studies. J Biosci.

[CR15] Kato M, Mizuno K (2004). Caffeine synthase and related methyltransferases in Plants. Front Biosci.

[CR16] Kozera B, Rapacz M (2013). References genes in real-time PCR. J Appl Genet.

[CR17] Kumar A, Giridhar P (2015). Salicylic acid and methyl jasmonate restore the transcription of caffeine biosynthetic *N*-methyltransferases from a transcription inhibition noticed during late endosperm maturation in coffee. Plant Gene.

[CR18] Kumar A, Simmi PS, Naik GK, Giridhar P (2015). RP-HPLC and transcript profile indicate increased leaf caffeine in *Coffea canephora* plants by light. J Biol Earth Sci.

[CR19] Kumar A, Naik GK, Simmi PS, Giridhar P (2015). Salinity and drought response alleviate caffeine content of young leaves of *Coffea canephora* var. Robusta cv. S274. J Appl Biol Biotechnol.

[CR20] Kumar A, Naik GK, Giridhar P (2017). Dataset on exogenous application of salicylic acid and methyl jasmonate and the accumulation of caffeine in young leaf tissues and catabolically inactive endosperms. Data Brief.

[CR21] Livak KJ, Schmittgen TD (2001). Analysis of relative gene expression data using real time quantitative PCR and the 2^– ∆∆CT^ method. Methods.

[CR22] Perrois C, Strickler SR, Mathieu G, Lepelley M, Bedon L, Michaux S, Husson J, Mueller L, Privat I (2015). Differential regulation of caffeine metabolism in *Coffea arabica* (Arabica) and *Coffea canephora* (Robusta). Planta.

[CR23] Pfaffl MW, Tichopad A, Prgomet C, Neuvians TP (2004). Determination of stable housekeeping genes, differentially regulated target genes and sample integrity: BestKeeper–excel-based tool using pair-wise correlations. Biotechnol Lett.

[CR24] Pfaffl MW (2001). A new mathematical model for relative quantification in real-time RT-PCR. Nucl Acids Res.

[CR25] Ruijter JM, Pfaffl MW, Zhao S, Spiess AN, Boggy G, Blom J, Rutledge RG, Sisti D, Lievens A, de Preter K (2012). Evaluation of qPCR curve analysis methods for reliable biomarker discovery: Bias, resolution, precision, and implications. Methods.

[CR27] Silver N, Best S, Jiang J, Thein SL (2006). Selection of housekeeping genes for gene expression studies in human reticulocytes using real-time PCR. BMC Mol Biol.

[CR28] Vandesompele J, De Preter K, Pattyn F, Poppe B, Van Roy N, De Paepe A, Speleman F (2002). Accurate normalization of real-time quantitative RT-PCR data by geometric averaging of multiple internal control genes. Genome Biol.

[CR29] Vieira A, Talhinas P, Loureiro A, Duplessis S, Fernandez D, Silva M, Paulo C, Azinheira OS (2011). Validation of RT-PCR reference genes for *in planta* expression studies in *Hemileia vastatrix*, the causal agent of coffee leaf rust. Fungal Biol.

